# Accelerated early weight gain of neonatal puppies from overweight bitches compared to lean dams in spite of similar milk macronutrient composition

**DOI:** 10.3389/fnut.2026.1747221

**Published:** 2026-03-03

**Authors:** Samantha J. McCarter, Stephen R. Werre, Michael L. Power, Orsolya Balogh

**Affiliations:** 1 Virginia-Maryland College of Veterinary Medicine, Blacksburg, VA, United States; 2 Department of Population Health Sciences, Virginia-Maryland College of Veterinary Medicine, Blacksburg, VA, United States; 3 Smithsonian National Zoological Park and Conservation Biology Institute, Washington, DC, United States; 4 Department of Small Animal Clinical Sciences, Virginia-Maryland College of Veterinary Medicine, Blacksburg, VA, United States

**Keywords:** adiposity, dog, growth curve, milk composition, neonate, overweight, puppy

## Abstract

**Introduction:**

Lactation is the final pathway for the maternal metabolism to influence the neonate. Studies in women and animals have shown maternal body condition affecting milk composition and/or offspring growth and adiposity. The effect of the body condition of the dam on neonatal puppy growth rate has never been examined, despite a growing population of overweight breeding dogs. This study aimed to compare the milk macronutrient composition and puppy growth rate during the neonatal period between overweight and lean bitches.

**Methods:**

A total of 16 litters from 15 medium- to large-breed client-owned dogs were enrolled after whelping. Dams were classified into lean (LE, body condition score [BCS]: 4–5/9, *n* = 8) and overweight (OW, BCS: 6–7/9, *n* = 8) groups. Milk was collected from bitches at 5 timepoints (0.5, 1, 2, 3, and 4 weeks of lactation). Growth curves of a total of 106 puppies born alive to LE (*n* = 58) and OW (*n* = 48) mothers and exclusively nursing on the dam were analyzed until 21 days of age. Birth weight, daily body weights, average daily weight gain (ADGg), average daily percent gain (ADG%), average daily percent gain from birth (ADGB%), and total percent gain from birth (TGB%), as well as milk dry matter, crude protein, sugar, fat, ash, and calculated gross energy, were analyzed using a mixed-model analysis of variance (ANOVA); the significance level was set at a *p*-value of <0.05.

**Results:**

Puppy birth weights were unaffected by litter size and the dam BCS. Puppy growth curves and TGB% were significantly different between the two maternal groups. ADGg, ADG%, and ADGB% were significantly higher in OW dam puppies on day 2 and day 4 after birth. Puppies born heavier remained heavier, while ADG% and ADGB% were inversely related to birth weight. Litter size had no effect on these growth parameters. Swimmer puppy syndrome was observed in eight puppies from three OW dam litters. Dam BCS had no significant effect on milk macronutrients in the first 4 weeks of lactation.

**Discussion:**

In conclusion, despite the similar day-to-day neonatal body weights between the maternal groups, puppies from OW dams grew differently, gaining more weight in the first week of life. Factors other than milk macronutrient composition are likely responsible for these differences.

## Introduction

Obesity is a global health problem in humans and pets. In 2022, the U.S. Pet Obesity Prevalence Survey found that 59% of dogs were classified as overweight or obese by their veterinarians (1). There is a growing population of overweight and obese bitches being bred (2), especially considering show standards promote an over-conditioned body type in some breeds such as Molossoids, Swiss Mountain and Cattle Dogs, Labrador Retrievers, Water Dogs, Pugs, and Basset Hounds (3, 4). There are numerous studies that have identified adverse health effects of obesity on lifetime health and longevity in dogs (5, 6), such as increasing the risk of insulin resistance (7), altered cardiac structure and function (8), and orthopedic disorders (9). Despite this body of literature, there are still no clearly documented studies on the effects of increased adiposity on the reproductive performance of female dogs, including milk composition in nursing dams and the resulting neonatal health outcomes.

Lactation is the final pathway for the maternal metabolism to influence the neonate, with studies supporting obesity having a negative impact on lactation (10, 11). Overweight and obese women are less likely to initiate lactation, have a delayed onset of lactogenesis II (copious milk production), and have a generally shorter duration of breastfeeding (12, 13), although the exact reason is poorly understood. Overweight and obese mothers have also reported that their infants suckled for longer durations, and they had reduced prolactin responses to suckling at the first 48 h of lactation (14). In sows, obese dams had a higher occurrence of postpartum dysgalactia syndrome, hypothesized to be from an increased catabolic rate from higher maternal fat deposits (15). In dogs, obesity was mentioned as a potential contributor to increased neonatal puppy mortality, in part due to hypogalactia (16, 17); however, there has not been a controlled study to provide clear evidence. The reproductive consequences of canine maternal overweight and obesity, including milk production and composition and neonatal health, are largely unknown (18).

Canine milk is typically higher in protein and fat than human, bovine, or caprine milk (19–21). Milk composition changes in healthy bitches throughout lactation, with some disagreements between the studies. Over the first month of lactation, the total protein of canine milk was shown to either decline (22) or remain unchanged (19), total lipids were unchanged (19, 22), and there was an increase in lactose through the first week (23) and month of lactation (19, 22). Gross energy either increased in the first week (23) or remained unchanged throughout the first month of lactation (19, 22). Additionally, one study found subtle differences in milk composition across dog breeds of similar sizes (21), while another study found no difference between breeds of different sizes (24).

Maternal adiposity has been shown to affect milk composition, although the results are inconsistent. In women, several studies have found that breast milk of obese mothers had higher fat (25–27), calories (27), and protein content (25). In contrast, other studies have described no difference in the overall breast milk fat content (28, 29) despite significant differences in the long-chain fatty acid profiles (28–31), such as increased n-6/n-3 polyunsaturated fatty acids and decreased docosahexaenoic acid (28–31). These changes in altered fatty acid profiles were subsequently shown to be associated with increased infant body mass index (BMI) and weight at birth (28, 29, 31), 2 months (31), 6 months (30), and up to 13 months (29), although these associations were found to generally disappear as children aged (28, 30, 31). Additionally, breast milk of obese mothers also had significantly higher levels of triglycerides (27), leptin (32, 33), and insulin (27, 28, 32, 33), which affected the neonatal intestinal microbiome without having a significant impact on infant weight gain or body fat percentage at 2 weeks of age (33). Despite the large breadth of research in this field, a systematic review concluded that the majority of human studies were too variable and many were of low quality (26).

The impact of maternal adiposity on offspring growth has also been studied in other species. The litter average pre-weaning growth rate was higher in over-conditioned sows despite lower predicted milk yield in the second week of lactation than in under-conditioned sows (15). In mice, offspring from obese dams were hyperphagic from 4 to 6 weeks of age and eventually had increased abdominal fat mass at 3 months and body weight at 6 months compared to control offspring (34). To establish the effect of lactation alone, other murine studies used cross-fostering, i.e., pups born to lean mothers were nursed on obese dams. These fostered pups had increased caloric intake and ended up having higher body weight, body fat, and plasma insulin and leptin concentrations (35, 36). However, one study has only found a sex-dependent increase in perirenal fat (37). Kittens of overweight queens grew faster after weaning and were heavier at 5–6 months of age than kittens from lean dams or from dams whose non-pregnant body condition fluctuated between overweight and lean (38). While normal growth curves have been established for the average neonatal puppy (39) and between different breeds and sizes (40–43), it is yet unknown how maternal adiposity in dogs affects milk composition and early puppy growth.

This study aimed to compare the milk macronutrient composition and puppy growth rate during the neonatal period between overweight and lean bitches. We hypothesized that maternal over-conditioning will result in higher milk fat content and increased milk gross energy, and, in turn, these changes may lead to increased body weight gain in the puppies during the exclusive nursing period (from parturition to day 21 postpartum) compared to lean bitches.

## Materials and methods

All experimental procedures were approved by the Institutional Animal Care and Use Committee of Virginia Tech (Protocol #22-032). Informed consent was obtained from owners of all participating dogs.

### Animals

**Figure 1:**
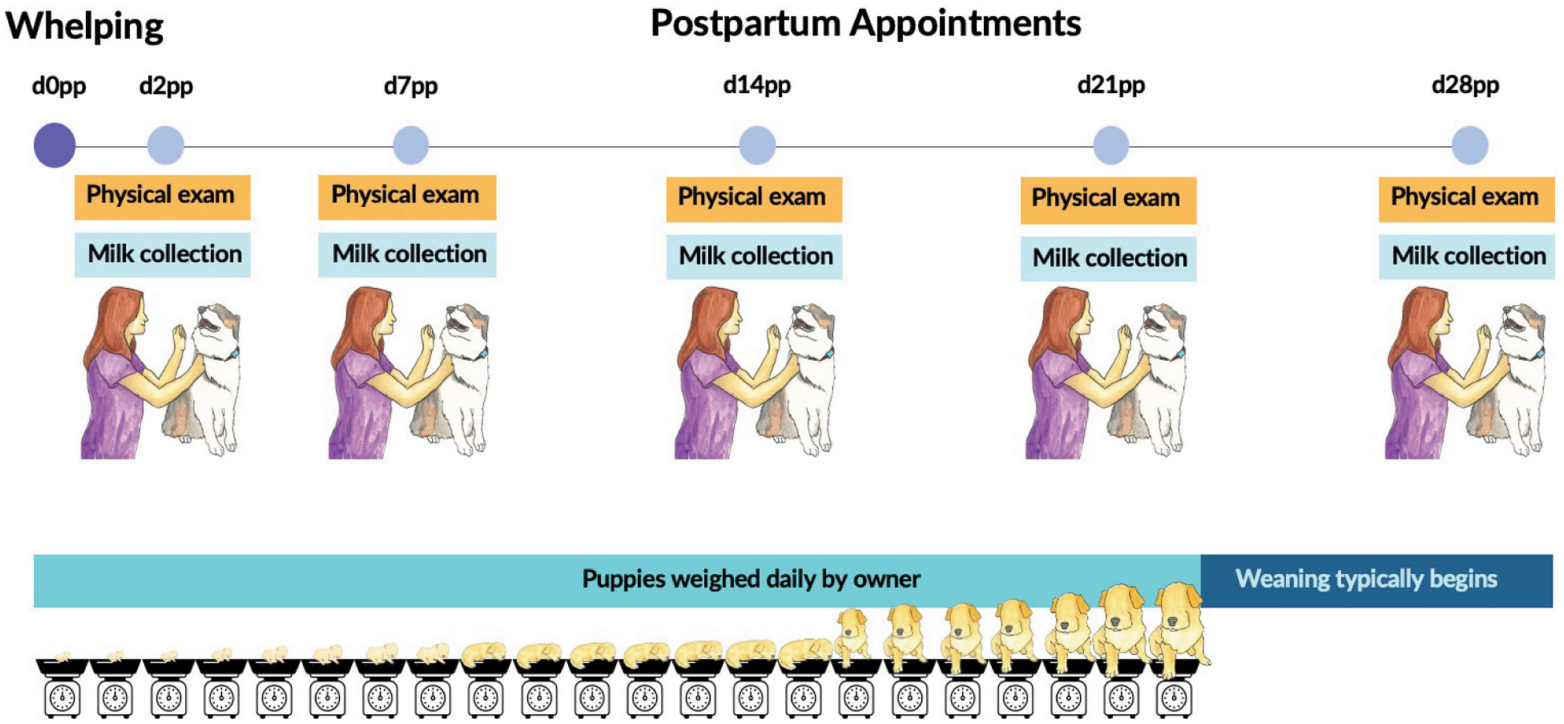
Experimental design and sampling timeline of the study. Days (d) are depicted postpartum (pp; d0 is the day of whelping).

A total of 15 client-owned, healthy, medium- to large-breed bitches were enrolled in the study for one lactation period, except for one bitch that was enrolled for two consecutive lactations (study participants characteristics are shown in Table 1). Dogs were housed in their owners’ homes with their puppies throughout the study. The bitches were examined by a veterinarian and confirmed healthy at the time of enrollment and throughout the study; see experimental design in Figure 1. Dams were grouped into lean (LE; body condition score [BCS]: 4–5/9, *n* = 8) and overweight (OW; BCS: 6–7/9, *n* = 8) categories based on their BCS [1–9 point scale (44)] at enrollment. Dogs were confirmed to be fed a complete and balanced commercial diet before parturition and were kept on the same diet throughout lactation except for one dog. During lactation, 12 bitches were fed the same commercial, dry, all-life-stages diet from a major pet food brand, and the remaining were fed all-life-stages or puppy dry food from three other different brands. A variety of treats and top dressings were also fed to stimulate appetite. Bitches were fed according to daily energy requirements (DER, kcal/day) calculated using current guidelines (45), according to the week of lactation, litter size, and current body weight.

**Table 1:** General demographics of the participating bitches.

Animal ID	BCS group	Body weight (kg) (pre/post)	Age (year)	Previous litters	Breed	Litter size	Comments
1	LE	32.9/31.9	3.6	0	Labrador Retriever	6	
2	LE	21.8/27.6	5.0	1	Australian Shepherd	7^−2^	
3	LE	24.5/26.9	2.5	0	Doberman Pinscher	7	^§^Not included in puppy growth analysis
4	LE	30.4/30.9	2.4	0	Labrador Retriever	8	
5	LE	22.3/25.8	2.9	0	Golden Retriever	8	Cesarean section
6	LE	NA / 25.8	4.4	1	Weimaraner	9	
7	LE	25.8/29.1	3.5	0	Weimaraner	11	
8	LE	33.0/38.6	2.2	0	Labrador Retriever	11**	Mastitis on day 20 postpartum
9	OW	29.6/28.1	3.9	0	Golden Retriever	3	Cesarean section, ^§§^Not included in puppy growth and milk analysis
10	OW	NA / 24.0	2.6	0	English Shepherd	5	
11	OW	27.0/30.8	5.3	1	Golden Retriever	6*	Cesarean section for the last three pups
12	OW	33.0/29.2	4.0	0	Labrador Retriever	7^−1^	
13	OW	NA/20.8	3.4	1	Australian Shepherd	7^−1^	
8	OW	35.5/39.4	3.1	1	Labrador Retriever	8^−1^	In the LE group for previous litter
14	OW	30.0/29.2	4.9	2	English Shepherd	8^‡^	
15	OW	23.6/29.8	5.1	2	Weimaraner	11^−1^*	

*One puppy died on day (d)1 after birth. **One puppy died on d2 after birth. ^‡^One puppy required milk supplementation from d15 onward. ^-n^ Number of stillborn puppies born, removed from litter size count. ^§^Puppy/ litter growth data not included in analysis due to substantial missing data. ^§§^Milk and puppy/litter growth data not included in analysis due to rapid body condition changes of the dam immediately postpartum. BCS = body condition score, LE = lean BCS (4–5/9), OW = overweight BCS (6–7/9). Body weights are given as measured during estrus breeding management (pre) and 3 weeks postpartum (post); NA: not available.

### Monitoring of puppy growth

All puppies in the litter of each bitch were weighed daily by their owners at the same time of day from birth (d0) to 21 days of age (Figure 1). Puppies were examined at the first study appointment to screen for congenital abnormalities and general health and then visually examined at subsequent visits. Puppies that became ill and needed veterinary medical attention and treatment or had supplemental feeding with a canine milk replacer were excluded from further data collection.

### Milk sampling

Milk was collected from the dams on days (d) 2–5, 7–11, 13–17, 20–26, and 27–33 after parturition (Figure 1) for milk macronutrient analysis. Puppies were separated from their mothers for a variable time (0–2 h) before sampling, and no medications were given to facilitate milk letdown. The teats of the bitches were disinfected with alcohol pads, the initial milk stream was discarded, and milk was collected into sterile plastic microcentrifuge tubes without preservatives from all expressible mammary glands to obtain a mixed sample (0.5–2.0 mL/bitch/day). The ease of milk collection varied between bitches, and the mammary glands were not fully milked out. The milk samples were kept on ice for 1–2 h before freezing and storing at −80 °C until the batch analysis.

Bitches were excluded from the study if they developed mastitis, metritis, or became severely ill for any other reason. Dystocia and Cesarean section were not disqualifying criteria unless complicated by metritis or a macerated fetus.

### Milk macronutrient analysis

All milk macronutrient assays were performed at the Smithsonian National Zoological Park Nutrition Laboratory using standard methods previously developed on location (46). Dry matter, fat, total sugar, crude protein (CP), and total mineral content (ash) of the milk samples were measured in duplicate or triplicate.

The samples were homogenized by vortexing before they were subsampled for procedures. Dry matter, and therefore water content, was determined gravimetrically by weighing samples to 0.001 mg before and after a 3.5-h drying period at 100 °C in a forced air-drying oven (47). The dried samples were combusted in a carbon, hydrogen, and nitrogen elemental gas analyzer (EMA 502 Elemental Analyzer CHNS-O, VELP, Deerpark, NY or Model 2,400, Perkin Elmer, Norwalk, CT) to determine total nitrogen (TN) content. Total fat was measured using a micro-modification of the Rose–Gottlieb procedure that involves sequential lipid extraction with ethanol, diethyl ether, and petroleum ether (46). Total sugar was measured using the phenol- sulfuric acid colorimetric procedure with lactose monohydrate standards and was read at 490 nm on a microplate reader (Model ELX808, BioTek, Winooski, VT) (48, 49). Ash was measured by placing the dried milk samples in a muffle furnace at 550 °C for 8 h. Milk gross energy (GE) was calculated for each sample as the sum of the energy from protein, fat, and sugar, using the following energy values: 5.86 kcal/g for protein, 9.11 kcal/g for fat, and 3.95 kcal/g for sugar (50). This method of GE calculation may be a slight overestimate because it does not account for non-protein nitrogen. However, this calculated GE value has been shown to closely correlate with experimentally measured GE using adiabatic bomb calorimetry for milk from species as diverse as aardvarks [*Orycteropus afer* (51)], bongos [*Tragelaphus eurycerus* (52)], and rhesus macaques [*Macaca mulatta* (53)].

The content of each macronutrient is reported as a percent of the weight of milk (g/100 g). The calculated GE of each sample was used to calculate the percentage of GE from sugar, fat, and protein (mg/kcal GE).

### Statistical analysis

For puppy growth, individual puppy body weight data from birth to 21 days of age was evaluated, as this was the period when all puppies were exclusively nursing on the dams and nutritional weaning had not yet started. The puppies of bitch #9 were excluded from the analysis because of rapid weight and BCS changes of the dam due to gastrointestinal illness postpartum. Due to substantial missing data, puppies of bitch #3 were also excluded from the analysis. One puppy from the litter of an OW bitch was excluded from the analysis from d15 onward (bitch #14, Table 1) because the owner started supplemental bottle feeding of this one puppy. Data from the litter of an LE bitch (#7) were missing from d17 onward. Small gaps (1–3 days) in missing data were estimated using the previous and next day’s weights. Stillborn puppies were not counted for litter size and not weighed by the owners. Litter size was adjusted for deceased puppies for each day analyzed.

The following variables were calculated from daily individual puppy body weight data to represent growth parameters that are clinically used to monitor puppy growth rate independent of breed: average daily weight gain (ADGg), average daily percent gain (ADG%), average daily percent gain from birth (ADGB%), and total percent gain from birth (TGB%). Additionally, several litter-level variables, i.e., litter average daily percent gain (LiADG%), litter average daily percent gain from birth (LiADGB%), and litter total percent gain from birth (LiTGB%), were also calculated. All equations are shown in Table 2.

**Table 2:** Calculation of parameters used for individual puppy and litter growth statistical analysis.

Growth measurement	Equation
ADGg	[CurrentBW]−[PreviousBW]
ADG%	$\frac{\left[\text{Current}\;\mathrm{BW}]-[\text{Previous}\;\mathrm{BW}\right]}{\left[\text{Previous}\;\mathrm{BW}\right]}\times 100$
LiADG%	$\frac{\left[\text{Litter current total}\;\mathrm{BW}]-[\text{Litter previous total}\;\mathrm{BW}\right]}{\left[\text{Litter previous total}\;\mathrm{BW}\right]}\times 100$
ADGB%	$\frac{\left[\text{Current}\;\mathrm{BW}]-[\text{Previous}\;\mathrm{BW}\right]}{\left[BW_{0}\right]}\times 100$
LiADGB%	$\frac{\left[\text{Litter current total}\;\mathrm{BW}]-[\text{Litter previous total}\;\mathrm{BW}\right]}{\left[\text{Litter total}\;BW_{0}\right]}\times 100$
TGB%	$\frac{\left[\text{Current}\;\mathrm{BW}]-[BW_{0}\right]}{\left[BW_{0}\right]}\times 100$
LiTGB%	$\frac{\left[\text{Litter current total}\;\mathrm{BW}]-[\text{Litter total}\;BW_{0}\right]}{\left[\text{Litter total}\;BW_{0}\right]}\times 100$

BW, puppy body weight (grams); BW_0_, birth weight (grams); ADGg, average daily weight gain in grams; ADG%, average daily percent gain; LiADG%, litter average daily percent gain; ADGB%, average daily percent gain from birth; LiADGB%, litter average daily percent gain from birth; TGB%, total percent gain from birth; LiTGB%, litter total percent gain from birth.

Individual puppy birth weight was analyzed using a mixed-model ANOVA, with litter identification as the subject of repetition and litter size and the maternal BCS group (LE vs. OW) as the fixed effects. Individual puppy growth curves (i.e., daily body weights), ADGg, ADG%, ADGB%, TGB%, LiADG%, LiADGB%, and LiTGB% were analyzed using a mixed-model ANOVA, where puppy identification nested within litter identification (for individual-level models) and litter identification (for individual-level and litter-level models) was included as the subject of repetition, individual puppy birth weight or litter average birth weight (for litter-level calculations) as covariate, and litter size, time (day after birth), maternal BCS group, and the interaction term time x maternal BCS group as fixed effects. The Tukey–Kramer test was used for adjusting for multiple comparisons in all models.

Owing to the slight variations in milk sampling times due to logistics and owner availability, the following weekly timepoints were used for statistical comparisons of milk components: week 0.5 (d2–5), week 1 (d7–11), week 2 (d13–17), week 3 (d20–26), and week 4 (d27–33). Milk macronutrients, i.e., fat (%, g/100 g milk), CP (%, g/100 g milk), sugar (%, g/100 g milk), ash (%, g/100 g milk), and estimated GE (kcal/g), were analyzed using a mixed-model ANOVA, with litter identification as the subject of repetition and litter size, time, maternal BCS group (LE vs. OW), and the interaction term time x maternal BCS group as fixed effects. Bitch #9 (Table 1) was also excluded from the milk analysis. Milk collection was discontinued for bitch #8 when she developed mastitis on d20 pp.

For all analyses, the significance level was set at a *p*-value of < 0.050. Data are shown as least squares means and standard error (SE) unless specified otherwise. Analyses were performed using Statistical Analysis Software (SAS) version 9.4 (Cary, NC, USA).

## Results

Three bitches required cesarean sections due to dystocia. One bitch (#8) was included for two separate litters; she was lean during her first lactation and overweight 10 months later during her second lactation (Table 1).

Across the 16 litters in our study, 116 puppies were born alive (*n* = 68 from LE dams; *n* = 48 from OW dams), and 7 were stillborn (*n* = 2 from LE dams; *n* = 4 from OW dams). After eliminating bitches #3 and #9 from the puppy growth analysis, 106 puppies remained (*n* = 58 from LE dams; *n* = 48 from OW dams). Three puppies from different litters died within the first week.

### Neonatal puppy growth

#### Birth weight

**Figure 2:**
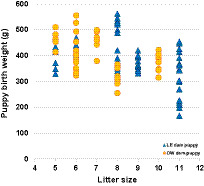
Puppy birth weight by litter size and maternal groups. LE, lean; OW, overweight.

Individual puppies were born with a mean birth weight of 378.4 g (range: 167.3–561.3 g) in LE dams and 406.4 g (range: 255.1–555.7 g) in OW dams. The dam BCS group and litter size had no impact on birth weight (*p* = 0.954 and *p* = 0.127, respectively) (Figure 2).

#### Puppy growth curves

By 21 days of age, the body weight of the puppies increased to an average of 1581.8 g (range: 1083.0–2040.0 g) in LE dams and 1809.3 g (range: 1221.3–2545.8 g) in OW dams.

**Figure 3:**
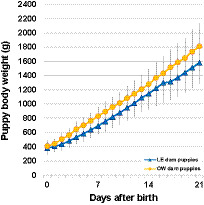
Growth curves depicting puppy daily body weights over time and by maternal groups. Data points are shown as means, and whiskers are shown as standard deviation.

Puppy growth curves, based on day-to-day body weights (g), were significantly affected by both time and the maternal BCS group (*p* < 0.0001) despite puppy weights not being significantly different on any given day between OW and LE mothers (Figure 3). Litter size had no effect on individual puppy growth curves (*p* = 0.447), while puppies with higher birth weight remained heavier throughout the study (*p* < 0.0002).

#### Puppy average daily weight gain (ADGg)

**Figure 4:**
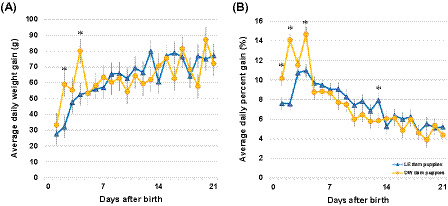
**(A)** Individual puppy average daily weight gain (ADGg) and **(B)** average daily percent gain (ADG%) over time and by maternal groups. Data points are shown as least squares means, and whiskers are shown as SE. The asterisks denote significant differences between puppies from overweight (OW) and lean (LE) dams (*p* < 0.05).

Individual puppy ADGg ranged from −181.4 to 260.0 g in LE dams and −102.1 to 226.8 g in OW dams during the first 21 days after birth. ADGg increased over time in both groups, with a significant interaction between time and the maternal BCS group (*p* < 0.0001; Figure 4A). ADGg was higher in puppies of OW dams on d2 and d4 by an average of 26.8 g and 27.4 g, respectively (*p* ≤ 0.012). Litter size and birth weight had no effect on ADGg (*p* = 0.362 and *p* = 0.102, respectively).

#### Puppy average daily percent gain (ADG%) and litter average daily percent gain (LiADG%)

Individual puppy ADG% ranged from −16.7 to 44.1% in LE dams and −17.1 to 48.6% in OW dams during the first 21 days after birth. ADG% decreased over time in both groups, with a significant interaction between time and the maternal BCS group (*p* < 0.0001). ADG% was higher in puppies of OW dams on d1, d2, and d4 (2.5, 6.5, and 3.7%, respectively, *p* ≤ 0.014), while LE dams’ puppies gained more on d13 (2.1%, *p* = 0.048) (Figure 4B). Litter size had no effect on ADG% (*p* = 0.338); however, the birth weight had a small but significant (*p* < 0.0001) effect. Heavier-born puppies gained proportionally slightly less each day throughout the first 21 days after birth, i.e., a puppy that was born 10 g heavier gained an average of 0.10% less per day.

LiADG% ranged from −1.4 to 17.4% in LE dams and −3.5 to 28.7% in OW dams during the first 21 days after birth. LiADG% decreased over time (*p* < 0.0001), while the maternal BCS group or its interaction with time, or litter size had no effect (*p* ≥ 0.101). Litter average puppy birth weight had a small but significant inverse relationship with LiADG% (*p* = 0.009).

#### Puppy average daily percent gain from birth (ADGB%) and litter average daily percent gain from birth (LiADGB%)

**Figure 5:**
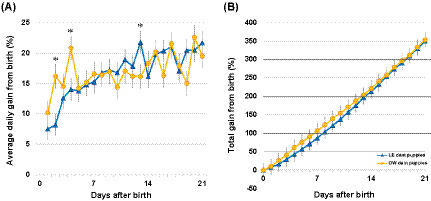
**(A)** Individual puppy average daily percent gain from birth (ADGB%) and **(B)** total percent gain from birth (TGB%) over time and by maternal BCS groups. Data points are shown as least squares means, and whiskers are shown as SE. The asterisks denote significant differences between puppies from overweight (OW) and lean (LE) dams (*p* < 0.05). Bolded lines indicate when weight doubled (100%), tripled (200%), and quadrupled (300%) from birth.

Individual puppy ADGB%, that is, daily weight gain as a percentage of birth weight, ranged from −50.0 to 72.2% in LE dams and −33.6 to 71.1% in OW dams during the first 21 days after birth. ADGB% increased over time in both groups, with a significant interaction between time and the maternal BCS group (*p* < 0.0001; Figure 5A). ADGB% was higher in puppies of OW dams on d2 and d4 (8.0 and 6.8%, respectively, *p* ≤ 0.013), while LE dams’ puppies proportionally gained more on d13 (5.7%, *p* = 0.040) (Figure 5A). Litter size had no effect on ADGB% (*p* = 0.337), while the birth weight had a small but significant (*p* < 0.0001) effect. Heavier-born puppies gained slightly less throughout the first 21 days after birth, i.e., a puppy that was born 10 g heavier gained an average of 0.42% less per day.

LiADGB% ranged from −1.3 to 40.2% in LE dams and −3.5 to 40.8% in OW dams during the first 21 days after birth. LiADGB% increased over time after birth (p < 0.0001), while the maternal BCS group or its interaction with time, or litter size had no effect (*p* ≥ 0.163). Litter average puppy birth weight had a small but significant inverse relationship with LiADGB% (*p* = 0.008).

#### Puppy total percent gain from birth (TGB%) and litter total percent gain from birth (LiTGB%)

By 21 days after birth, TGB% increased to a mean of 337.2% (range: 162.5–601.5%) in LE dams and 354.2% (range: 175.0–746.8%) in OW dams. TGB% was significantly affected by the dam BCS group over time (*p* = 0.002). Puppies from OW dams doubled their birth weight by d7 compared to LE dam puppies reaching this milestone by d8. On average, all puppies tripled and quadrupled their birth weight by d13 and d19, respectively (Figure 5B). Litter size and birth weight had no impact on TGB% (*p* = 0.600 and *p* = 0.126, respectively).

By 21 days after birth, LiTGB% increased to a mean of 334.7% (range: 201.1–430.5%) in LE dams and 348.7% (range: 206.9–513.6%) in OW dams. LiTGB% was not affected by the dam BCS group or its interaction with time (*p* ≥ 0.748). As expected, LiTGB% increased over time (*p* < 0.0001), with mean litter weights doubling by d7–8, tripling by d13, and quadrupling by d19. Litter size had a significant effect (*p* = 0.003), indicating that larger litters gained less, i.e., an increase of one puppy in the litter resulted in a 21.5% lower LiTGB% over time. Litter average puppy birth weight had a significant inverse relationship with LiTGB% (*p* = 0.003).

#### Mobility developmental abnormalities of the puppies

Within the second week after birth, mild swimmer puppy syndrome was diagnosed in eight puppies from three different litters of OW dams (litter #9 on d8, all 3 puppies affected; #11 on d10, 2 puppies affected out of 5; and #12 on d14, 3 puppies affected out of 6; Table 1) despite adequate husbandry and an environment providing good footing. Diagnosis by the investigators was made based on dorsoventrally flattened chests and caudolaterally splaying hind legs when ambulating. Intervention was immediately implemented through limiting affected puppies’ total daytime nursing time on the dam to decrease daily weight gain, physical therapy, and hobbling until all puppies were able to walk without deficits. When affected pups’ weights were examined more closely, those within only partially affected litters (#11 and #12) were not all born the heaviest. The mean week 1 ADG% among swimmer puppies was 9.7% (range: 9.0–10.7%) in litter #9 and 11.0% (range: 10.2–11.8%) in litter #11. In litter #12, the week 1 and week 2 mean ADG% of swimmer puppies was 12.5% (range: 11.9–13.3%) and 6.5% (range: 5.9–7.5%), respectively. Non-affected puppies in litter #11 had a mean ADG% of 10.5% (range: 9.2–11.7%) in week 1, while non-affected puppies in litter #12 had a mean ADG% of 12.4% (range: 11.8–13.1%) and 6.9% (range: 6.2–8.2%) during weeks 1 and 2, respectively. All of these values exceeded the mean ADG% of LE dam puppies during weeks 1 and 2 but were similar to the other OW litters.

### Milk composition

#### Milk dry matter (DM, g/100 g)

**Figure 6:**
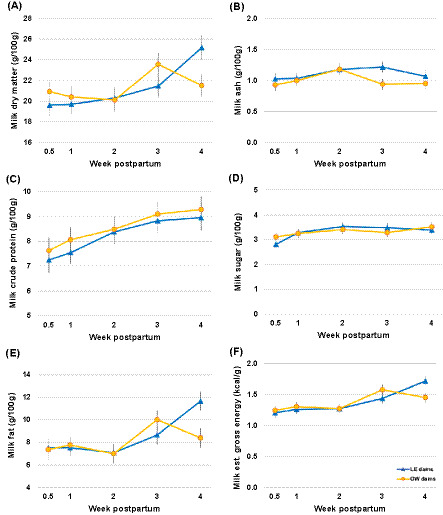
Milk macronutrients over time and by maternal groups. Data points are shown as least squares means, and whiskers are shown as SE in the overweight (OW) and lean (LE) dams. **(A)** Milk dry matter, **(B)** Milk ash, **(C)** Milk crude protein, **(D)** MIlk sugar, **(E)** Milk fat, **(F)** Milk estimated gross energy.

The mean milk DM during the first 4 weeks of lactation was 20.9% (g/100 g milk; range: 16.9–27.0%) in LE dams and 21.3% (range: 16.5–27.3%) in OW dams. Dam BCS group and litter size had no effect on milk DM (*p* ≥ 0.069). Time had a significant influence, and DM increased from early lactation to week 4 (*p* = 0.004) (Figure 6A; Table 3).

**Table 3:** Milk macronutrients in overweight (OW) and lean (LE) dogs over time.

Milk macronutrient	BCS Group	Weeks postpartum				
		0.5	1	2	3	4
Dry matter	LE	19.6 ± 1.1	19.7 ± 0.9	20.3 ± 1.0	21.4 ± 1.1	25.2 ± 1.2
(g/100 g)	OW	20.9 ± 1.2	20.4 ± 1.2	20.1 ± 1.1	23.6 ± 1.1	21.5 ± 1.1
	Total	20.3 ± 0.8^a^	20.0 ± 0.7^a^	20.2 ± 0.7^a^	22.5 ± 0.8^ab^	23.3 ± 0.8^b^
Ash	LE	1.0 ± 0.1	1.0 ± 0.1	1.2 ± 0.1	1.2 ± 0.1	1.1 ± 0.1
(g/100 g)	OW	0.9 ± 0.1	1.0 ± 0.1	1.2 ± 0.1	0.9 ± 0.1	1.0 ± 0.1
	Total	1.0 ± 0.1	1.0 ± 0.1	1.2 ± 0.1	1.1 ± 0.1	1.0 ± 0.1
Crude protein	LE	7.2 ± 0.5	7.5 ± 0.5	8.4 ± 0.5	8.8 ± 0.5	9.0 ± 0.5
(g/100 g)	OW	7.6 ± 0.5	8.1 ± 0.5	8.5 ± 0.5	9.1 ± 0.5	9.3 ± 0.5
	Total	7.4 ± 0.4^a^	7.8 ± 0.3^a^	8.4 ± 0.4^ab^	9.0 ± 0.4^b^	9.1 ± 0.4^b^
Sugar	LE	2.8 ± 0.2	3.3 ± 0.2	3.5 ± 0.2	3.5 ± 0.2	3.4 ± 0.2
(g/100 g)	OW	3.1 ± 0.2	3.3 ± 0.2	3.4 ± 0.2	3.3 ± 0.2	3.5 ± 0.2
	Total	3.0 ± 0.1^a^	3.3 ± 0.1^ab^	3.5 ± 0.1^b^	3.4 ± 0.1^ab^	3.5 ± 0.1^b^
Fat	LE	7.5 ± 0.8	7.5 ± 0.7	7.1 ± 0.8	8.7 ± 0.8	11.7 ± 0.8
(g/100 g)	OW	7.4 ± 0.8	7.8 ± 0.8	7.0 ± 0.8	10.0 ± 0.8	8.4 ± 0.8
	Total	7.4 ± 0.6^a^	7.7 ± 0.5^a^	7.0 ± 0.6^a^	9.3 ± 0.6^ab^	10.0 ± 0.6^b^
Est. GE	LE	1.2 ± 0.1	1.3 ± 0.1	1.3 ± 0.1	1.4 ± 0.1	1.7 ± 0.1
(kcal/g)	OW	1.2 ± 0.1	1.3 ± 0.1	1.3 ± 0.1	1.6 ± 0.1	1.5 ± 0.1
	Total	1.2 ± 0.1^a^	1.3 ± 0.1^a^	1.3 ± 0.1^a^	1.5 ± 0.1^b^	1.6 ± 0.1^b^
%GE from crude protein	Total	35.4%	35.5%	38.8%	35.0%	33.7%
%GE from sugar	Total	9.7%	10.1%	10.9%	8.9%	8.7%
%GE from fat	Total	55.0%	54.4%	50.3%	56.1%	57.6%

Results are shown as least squares mean ± SE. Different superscripts in a row denote a significant difference between weeks postpartum (*p* < 0.05). No differences between OW and LE groups were found. BCS: body condition score.

#### Milk ash (g/100 g)

The average ash content during the first 4 weeks of lactation was 1.1% (g/100 g milk; range: 0.8–1.5%) in LE dams and 1.0% (range: 0.3–1.4%) in OW dams. Neither dam BCS group, time, nor litter size had an influence on milk ash (*p* ≥ 0.081; Figure 6B; Table 3).

#### Milk crude protein (CP, g/100 g)

The mean milk CP in LE dams was 8.0% (g/100 g milk; range: 5.8–11.4%) during the first 4 weeks of lactation and 8.5% (range: 6.4–11.8%) in OW mothers. The dam BCS group or litter size had no effect (*p* ≥ 0.557), while CP increased gradually over the first 4 weeks pp (*p* = 0.001) (Figure 6C; Table 3). The mean milk CP as a component of GE was 63.0 mg/kcal GE.

#### Milk sugar (g/100 g)

Both LE and OW dams had a mean milk sugar of 3.3% (g/100 g milk; range: 2.1–4.5% and 2.3–4.6%, respectively) during the first 4 weeks of lactation. Milk sugar increased significantly from week 0.5 to weeks 2 and 4 pp (*p* ≤ 0.029), independent of the dam BCS group or litter size (*p* ≥ 0.543; Figure 6D; Table 3). The mean milk sugar as a component of GE was 26.2 mg/kcal GE.

#### Milk fat (g/100 g)

LE and OW dams had a mean milk fat of 8.4 and 8.1% (g/100 g milk; range: 3.7–13.5% and 3.7–12.2%, respectively) during the first 4 weeks of lactation. Milk fat increased from early lactation to week 4 pp (*p* = 0.002), while the dam BCS group and litter size had no effect (*p* ≥ 0.093) (Figure 6E; Table 3). The mean milk fat as a component of GE was 57.9 mg/kcal GE.

#### Milk estimated gross energy (GE, kcal/g)

The mean estimated GE in the milk of LE and OW dams during the first 4 weeks of lactation was 1.4 kcal/g (range: 1.0–1.9 kcal/g and 0.9–1.8 kcal/g, respectively). GE increased from weeks 0.5–2 to weeks 3–4 (*p* ≤ 0.031), while it remained unaffected by the dam BCS and litter size (*p* ≥ 0.130) (Figure 6F; Table 3).

The greatest contribution to GE was from fat (mean 54.7%, range 50.3–57.6%), followed by crude protein (mean 35.7%, range 33.7–38.8%), and then sugar (mean 9.7%, range 8.7–10.9%). These values were relatively constant across lactation (Table 3).

## Discussion

This study is the first to examine the effect of increased canine maternal body condition on the neonatal puppy growth rate and milk macronutrient composition, despite its small sample size. Understanding the impact of over-conditioning of the dam during lactation while she is nursing her puppies and providing evidence-based results is essential to guide weight management recommendations for breeding broodbitches.

The dam’s BCS had no effect on puppy birth weight in our study population, although breeds were only partially matched across the maternal BCS groups. This finding is in agreement with feline (38), murine (34), swine (15), and one rat study (54), although the literature is not always consistent. Birth weights of rat pups born from obese dams were lower compared to normal-weight dams (37), but there was no difference in piglet litter weight at birth in over-conditioned sows (15). While the majority of human studies found no difference in infant birth weights from overweight or obese mothers (28, 30, 33, 55), several studies reported significantly increased weight-for-length z-score (28) or weight-for-age z-score (29, 31, 56).

Puppy growth curves (day-to-day body weights) were slightly but significantly affected by the dam BCS group during the first 3 weeks after birth. The literature is also divided and inconsistent about how maternal adiposity affects neonatal weights at different timepoints. Some studies comparing maternal adiposity levels have shown no weight differences in human infants after 2 weeks of age (28, 33), in female mouse pups before 3 months of age (34), or in piglet litter weight at weaning (15). However, a study in women has reported infant BMI and weight differences up to 2 months (31), and one cross-fostering study in rats has found lower birth weight, week 1 body weight, and week 2 body weight in pups nursing on obese dams (37). In contrast, another cross-fostering rat study has found pups from obese rats weighed more at 3 and 24 weeks of age with an increase in percent body fat (54). These cross-fostering studies have demonstrated that maternal adiposity exposure during lactation alone can be a significant determinant of offspring body weight and adiposity. The conflicting conclusions of maternal adiposity’s effect on pre-weaning neonatal weight gain between different species likely reflect many variables involved in growth, as well as differences between species, in experimental design, and maternal diet during lactation. The effects of maternal adiposity are also not limited to the early neonatal period. At 6 months of age, obese mouse dam pups had increased body weights, when hyperphagia was also observed, suggesting that the consequences of maternal adiposity may not emerge until post-weaning (34). When offspring were exposed to obese dams during lactation alone, they also had an increase in percent body fat at 6 months of age (54). We were unable to evaluate post-weaning puppy growth rate between the maternal groups in this study, although it would have been interesting to follow the puppies until adulthood.

Although dogs from more than one breed were included in this study, we selected participants for similar adult body weights. The percentage-based measurements (ADG%, ADGB%, and TGB%) were used to mitigate potential differences in puppy growth curves between the breeds. Nowadays, specific puppy growth charts are available for many dog breeds and for different adult weight classes (42, 43). Clinical practice guidelines also use a generally expected 5–10% daily weight gain for normal neonatal puppy growth rates in the first 3–4 weeks of life, independent of breed (39, 43). Our study has found that ADGg, ADG%, ADGB%, and TGB% were significantly affected by the interaction between the dam BCS and time, with OW dam puppies showing accelerated growth rates in the first 4 days of life. This finding was different from a study in kittens, which showed that kittens from OW queens did not have a significantly greater monthly weight until 4 months of age. However, that study followed kittens until 12 months of age and did not closely examine the changes during the neonatal period (38). A study in pigs has also found that piglets from higher BCS sows had a significantly higher mean daily body weight gain, although these litters also had significantly lower litter sizes compared to lower BCS sows, as piglet litter weights were not different at weaning (15). While our initial hypothesis that puppies from OW litters would be consistently heavier and grow faster was disproven, we did find that puppies from OW dams grew differently, especially during the first few days after birth.

We speculate that either our OW population’s adiposity was not significant enough to exert a more substantial effect, which may only become consistent at an obese maternal body condition (BCS 8–9/9), or the impact of an overweight condition would not be observable until later in a puppy’s life. Such long-term effects may not be detectable in such a small cohort of dogs and masked by many variables that affect body weight and body condition.

In our study, the maternal BCS had no effect on average daily percent gain at the litter level (i.e., LiADG% and LiADGB%) or total percent gain from birth (i.e., LiTGB%), most likely because of the lower statistical degrees of freedom from comparing litter averages instead of individual puppies.

Litter size did not have a significant effect on birth weight in this study. This is in contrast to previous canine literature showing that a larger litter size results in significantly lower individual birth weights (40, 57, 58), although one study found this significance only in medium- and larger-sized breeds (40). The most likely cause for this discrepancy may lie in the small sample size of our study and its limited range of litter sizes (range: 5–11). Litter size also had no effect on any individual puppy growth parameters; however, a significant inverse relationship with LiTGB% showed a 21.5% less total litter weight gain from birth with each additional puppy. Other canine studies have also found an inverse relationship between litter size and growth over time (40, 57, 58).

Birth weight had a positive relationship with puppy growth curves, indicating that puppies born heavier remained heavier overall. This finding is consistent with other studies evaluating puppy growth within the first (23, 40) and second week of life (39, 40), although birth weight was no longer a significant factor at week 3 after birth (40). In contrast, birth weight had no effect on ADGg as a representation of daily body mass gain (in grams), while there was an inverse relationship with ADG% and ADGB%, indicating that the percent daily weight change compared to the previous day or to birth weight was slightly but significantly less in puppies born heavier. This was also true at the litter level with increasing litter average puppy birth weight (LiADG%, LiADGB%, and LiTGB%). Our finding is similar to a human study that found large-for-gestational-age infants grew slowly enough to reach similar body mass to their appropriate-for-gestational-age-sized infant counterparts (59). Another study tracking the puppy growth rate in the first week of life has also found that puppies with lower than normal birth weight had higher growth rates after 2 days of age, which was attributed to compensatory growth (60).

We observed 8 of 116 live-born puppies develop mild swimmer puppy syndrome, all from OW dams and from 3 of the smallest litters with 3, 5, and 6 puppies. We suspect that incidents of swimmer syndrome, in spite of an adequately floored environment, may be related to excessive early weight gain before ambulation, which was seen on the whole study population level in the higher ADG% of OW dam puppies in the first week after birth compared to LE dam puppies despite no difference in ADGg. Interestingly, not all puppies in the same litter (except for litter #9 with a total of 3 puppies) were affected. This highlights that other factors, such as conformation and activity level of the individual puppies, may have played a role. Indeed, factors responsible for the development of swimmer syndrome in puppies have been studied before by Tomihari et al., who found that affected puppies in a guide dog colony were from significantly smaller litter sizes and had significantly higher body weights on days 10 and 28 after birth (61). Small litter size was also a significant risk factor found in a questionnaire study on 115 dogs (62) and in another report on 2,443 puppies (63). While some breed dispositions have been suspected, none have shown significance (63). These findings are unsurprising, as puppies from smaller litters are able to consume more milk with less competition. Further studies in a larger population of overweight/obese lactating bitches are needed to confirm our finding of maternal adiposity as a risk factor for swimmer puppy syndrome.

Milk macronutrients were not significantly affected by the dam BCS group over the first 4 weeks postpartum. Our current results suggest that canine milk macronutrient composition is well regulated and not influenced by maternal body composition. This is not the case in all species. Current human literature has found that breast milk from obese mothers contains either increased milk fat (25, 26) or calories (25). In rhesus macaques, one study has found that milk bioactive factors [e.g., epidermal growth factor (EGF) and transforming growth factor beta 2 (TGF-β2)] were significantly affected by maternal body mass (64). This study has also found correlations between these bioactive factors and milk energy through milk fat % and milk sugar %, though milk protein % was correlated with EGF only (64). It is possible that other milk components that were previously reported to be affected by maternal body composition in other species, such as milk fatty acids (28–31), metabolites (e.g., oligosaccharides and nucleotides) (55), and insulin or leptin (28, 33), could have affected puppy growth. Another explanation is that canine milk macronutrients are not affected by the dam’s BCS until they are obese (8–9/9 BCS), and our OW population was not over-conditioned enough to elicit a clear change. The majority of studies looking at other metabolically relevant milk components in an obese maternal group may give further insights. We were unable to determine milk production and the amount of milk consumed by puppies between OW and LE bitches, which could explain the differences in growth pattern. In clinical practice, puppy weight gain is considered the best estimate for maternal milk production, although this assessment encompasses the amount of milk consumed (not produced) along with all other milk components beyond macronutrients. In a research setting, turnover of body water of the puppies estimated from water kinetics by deuterium oxide administration can be used to estimate the volume of ingested milk and extrapolate it to quantify maternal milk production (19). However, this is impractical and impossible to perform on client-owned bitches.

Litter size did not have a significant impact on any milk components, which is contrary to the inverse relationship found between litter size and milk GE during the first week of lactation by Keller et al. (23). This difference may stem from the aforementioned study’s shorter sampling window or the current study’s limited range of litter sizes.

Milk DM significantly increased by week 4 after parturition. This pattern reflected the changes in milk fat and CP and was not seen in the only currently published longitudinal study’s reported values (19). Milk sugar also significantly increased over the first 4 weeks postpartum. This pattern is similar to that seen by Keller et al. in the first week postpartum (23) and is also in agreement with other canine studies that followed bitches until at least 4 weeks after birth (19, 22).

The changes in milk GE over time rightfully reflect that GE is calculated from milk CP, fat, and sugar, as it significantly increased over the first 4 weeks postpartum. This pattern is similar to the study of Keller et al. in the first week postpartum (23). In contrast to our findings, current canine longitudinal studies showed no difference in milk GE over the course of lactation (19, 22). This difference may be the result of dog breeds used (21) or a subtle contribution from the overweight bitches that only followed trends. On an energy basis, sugar’s contribution to GE decreased slightly toward the end with a corresponding slight increase in energy from fat. The contribution of crude protein to milk GE was relatively stable over time.

The numerical values of the different milk macronutrients, DM and GE, were either comparable or somewhat different from those published for canine milk, even when compared with samples collected at similar timepoints in lactation (19, 23). These discrepancies may be explained by differences in methodology, breeds, or husbandry (including nutrition) between studies. Furthermore, although we collected a homogenized milk sample from the available front and hind teats, we were unable to collect samples to standardize fore and hind milk within each gland or to standardize the timing of collection in relation to the puppies’ most recent nursing.

The main limitations of this study stem from using client-owned animals; however, this variety indeed represents a real-world population to which recommendations can be immediately applied. With our variety of six different dog breeds, dam weights ranged from 20.5 to 40.5 kg. A study by Zhang et al. has found that there can be breed-related differences in canine milk composition (21); however, their results are difficult to compare with our data since their sampling window was wide and many of these milk macronutrients do change throughout lactation. Puppy birth weights also ranged between 167.3 g and 561.3 g, and hence, we used several percentage-based alternative parameters (ADG%, ADGB%, and TGB%) to monitor and compare puppy weight gain. Growth curve equations have been developed to monitor canine neonatal puppies in the past, impacted significantly by breed, sex, or birth weight alone (39), but this single growth equation lost accuracy after day 12 when the growth of the puppies slowed during the neonatal period. Total milk production and consumption are clear unmeasured contributors to puppy growth; however, this parameter was not feasible to collect in our client-owned bitches due to a lack of standardization, variable teat conformation and thus feasibility to completely milk out the gland, and discouragement of unnecessary medications to stimulate milk let-down.

As with studies involving client-owned dogs, there are several variables beyond the control of the investigators, including but not limited to the owner’s home environment and the time owners are able to monitor puppies and ensure the dam is nursing them. For the health of the litters, minor interventions were advised to the owners. While puppies requiring milk supplementation were excluded from the data set, some puppies falling behind were advised to have more nursing time or access to fuller teats. The five diagnosed swimmer puppies from two OW litters were intervened on primarily by physical therapy and limiting total daytime nursing times. Litter #9 was excluded from the data analysis due to the intervention of significant nursing limitations and outlier litter size. While these minor interventions may have affected our results, owners and breeders would do these easy adjustments within their usual husbandry practices. Analyzed litter sizes ranged from 5 to 11 puppies, not lending to a full range of litter sizes; however, studying the effect of litter size was not the primary goal of the study. Having a more uniform litter size made our study population more homogenous and allowed for a more accurate determination of the impact of maternal body condition. As discussed previously, future studies would benefit from a larger sample size, a consistent diet, maternal groups with more distinct adiposity levels (i.e., lean vs. obese [BCS 8–9/9]), and the possibility of analyzing other milk biomarkers that have been shown to be affected by maternal adiposity in other species.

## Conclusion

Maternal overweight condition affected the growth curve of the puppies subtly but significantly. Puppies of over-conditioned dams gained significantly more during the first few days after birth. Birth weight was also a significant determinant; heavier-born puppies remained heavier overall while their percent daily gain was slightly less. Litter size did not affect individual puppy growth. Milk macronutrient composition did not differ between lean and overweight bitches, so milk consumption rather than milk macronutrient composition could have been the primary determinant of puppy and litter growth differences between the maternal groups. The role of other milk components not examined in the study cannot be ruled out. The small study sample size may have been a limiting factor in elucidating differences in milk macronutrient composition between overweight and lean bitches. Alternatively, differences in milk composition may not be seen until a truly obese maternal body condition (BCS 8–9/9) has been reached. Swimmer syndrome was diagnosed in a few puppies of overweight dams only, highlighting an important consequence of overweight maternal condition combined with small litter size on neonatal puppy health.

## Data Availability

The raw data supporting the conclusions of this article will be made available by the authors, without undue reservation.
